# ASRHR in Ethiopia: reviewing progress over the last 20 years and looking ahead to the next 10 years

**DOI:** 10.1186/s12978-022-01434-6

**Published:** 2022-06-13

**Authors:** Elsie Akwara, Kereta Worknesh, Lemessa Oljira, Lulit Mengesha, Mengistu Asnake, Emiamrew Sisay, Dagem Demerew, Marina Plesons, Wegen Shirka, Azmach Hadush, Venkatraman Chandra-Mouli

**Affiliations:** 1 Department of Sexual and Reproductive Health and Research/Human Reproduction Programme, World Health Organization, Geneva, Switzerland; 2 Adolescent and Youth Health and Development Programme, Pathfinder International, Addis Ababa, Ethiopia; 3 School of Public Health, College of Health, College of Health and Medical Sciences, Haramaya University, Addis Ababa, Ethiopia; 4 Youth Council, Talent Youth Association, Addis Ababa, Ethiopia; 5 Pathfinder International, Addis Ababa, Ethiopia; 6 Federal Ministry of Health, Addis Ababa, Ethiopia; 7 Ethiopia Get Up Speak Out Alliance, Addis Ababa, Ethiopia; 8 Child and Adolescent Health, World Health Organization, Addis Ababa, Ethiopia; 9 Health Systems Unit, World Health Organization, Lusaka, Zambia

## Abstract

Over the last two decades, improvements in Ethiopia’s socio-economic context, the prioritization of health and development in the national agenda, and ambitious national health and development policies and programmes have contributed to improvements in the living standards and well-being of the population as a whole including adolescents. Improvements have occurred in a number of health outcomes, for example reduction in levels of harmful practices i.e., in child marriage and female genital mutilation/cutting (FGM/C), reduction in adolescent childbearing, increase in positive health behaviours, for example adolescent contraceptive use, and maternal health care service use. However, this progress has been uneven. As we look to the next 10 years, Ethiopia must build on the progress made, and move ahead understanding and overcoming challenges and making full use of opportunities by (i) recommitting to strong political support for ASRHR policies and programmes and to sustaining this support in the next stage of policy and strategy development (ii) strengthening investment in and financing of interventions to meet the SRH needs of adolescents (iii) ensuring laws and policies are appropriately communicated, applied and monitored (iv) ensuring strategies are evidence-based and extend the availability of age-disaggregated data on SRHR, and that implementation of these strategies is managed well (v) enabling meaningful youth engagement by institutionalizing adolescent participation as an essential element of all programmes intended to benefit adolescents, and (vi) consolidating gains in the area of SRH while strategically broadening other areas without diluting the ASRHR focus.

## Introduction

This commentary takes stock of the progress made in adolescent sexual and reproductive health and rights (ASRHR) in Ethiopia in the last two decades. Firstly, it discusses the political, economic, social context and the policies and programmes, highlighting the enablers and barriers in addressing ASRHR. Secondly, it highlights levels and trends in key ASRHR indicators, paying attention to differing levels and trends in different groups of adolescents. Building on this it sets out an agenda for action.

The profile and distribution of adolescents has changed significantly over this period in Ethiopia with the estimated population of adolescents increasing from 15.6 million in 2000 to 26.8 million in 2020 [1]. Population growth means that declining rates of child marriage for example co-exist with higher absolute numbers of girls affected. This necessitates a holistic multisectoral approach to address their overall well-being as they transition into adulthood, leaving no one behind. Given that there are 10 years to the Sustainable Development Goals (SDG) target date, there is a pressing need to build on the lessons learnt in the 15 years of the Millennium Development Goals (MDG) era and the first 5 years of the SDGs era.

The aim of the commentary is therefore to review progress over the last two decades in selected ASRHR indicators, by addressing the following questions:

i. How have the national political, social and economic context as well as policies and programmes evolved with regards to ASRHR in the last two decades?
ii. In which health outcomes, harmful practices, health behaviours, health services use, and social determinants was there progress, and was the progress equitable and steady?
iii. What are the opportunities and challenges in moving ahead, and what key actions are needed to accelerate progress in the next 10 years?

## Over the last two decades, political, economic and social developments, and national health and development policies and programmes have contributed to the changes in ASRHR

### Improvements in the economic and social context

Ethiopia’s economic situation has improved in the last two decades. Since the early 2000s, Ethiopia’s economy has been one of the ten fastest growing in the world. From 2004 to 2013, the country’s average annual Gross Domestic Product (GDP) growth rate exceeded 10%, which was more than four percentage points higher than the average for Africa’s 26 other low-income countries [2]. According to data from 2004/2005, the poverty index stood at 38.7%, it declined to 29.6% in 2010/2011, and declined further to 27.8% in 2011/2012. The decline in poverty levels has been steeper in rural areas (30.4%) as compared to urban areas (25.7%) [2]. While this progress is uneven, Ethiopian children and adolescents as a whole are likely to be growing up in a context of declining poverty.

There has been impressive progress in primary school enrolment, but less progress in primary school completion, and in secondary attendance and completion. Further, the progress has been uneven in the country. The proportion of adolescents in Ethiopia with no education decreased by 70% between 2000 and 2016 and there was an increase of over 50% in those who have completed primary school in the same time period [3, 5]. While there has been an improvement in the net primary attendance, there is a high attrition rate with a significant decrease in school attendance at ages 14 and 15 [3–6]. Further, there is a significant urban–rural variation, with a higher proportion of adolescents living in urban areas having secondary education or higher over the last decade compared to their counterparts in the rural areas. Finally, new means of communication, including the use of social media, are transforming the lives of Ethiopian adolescents, while in this area there are disparities in access and use too [7].

### High-level commitment to health and development

In 1994, the International Conference on Population and Development (ICPD) launched its Plan of Action, which recognized that Sexual and Reproductive Health and Reproductive Rights (SRHR) are central to women’s rights and well-being and are important to achieving social and economic development. The Plan of Action lent legitimacy to local advocacy efforts, investment, action and research that improved SRHR at global, regional, national and local levels [8]. Since then, the Government of Ethiopia (GoE) has undertaken several measures to ensure that ASRHR is addressed in its work as part of the wider attention to SRHR. In 2000, Ethiopia organized the first signature event entitled “The National Youth Consultation workshop on Sexual and Reproductive Health” that brought together all stakeholders to discuss issues affecting youth and to develop a series of action plans regarding the country’s youth policies and development agendas [9]. Based on the momentum created by this event and a conducive environment to engage multiple stakeholders, the GoE initiated several national initiatives that would mainstream ASRHR into the national agenda.

### Contribution of national health and development policies and strategies

ASRHR has been mainstreamed into the national agenda through policies and strategies directly addressing unintended pregnancy and childbearing, maternal mortality, HIV/AIDS, child marriage, Female Genital Mutilation/Cutting (FGM/C), and violence against women and girls. This contributed to progress in a number of ASRHR outcomes as discussed in the subsequent section.

The GoE's national contraceptive provision and demand generation effort was strengthened through the development of its first National Reproductive Health strategy and its National Family Planning Guideline in 2006. Adolescents were a key population group to be addressed in these normative documents. In alignment with these documents, the GoE also developed a National Adolescent Sexual and Reproductive Health Strategy, which placed adolescent girls within the context of an overall effort targeting women and girls [10].

In aligning its agenda to the MDG target 5 on reducing maternal mortality, the GoE put in place a new strategy to strengthen the delivery of maternal health services in both clinic and outreach settings through Health Extension Workers (HEWs), a category of health care providers used to provide care for a broad range of health issues [11, 12]. A focus of this strategy was improving access to quality antenatal care (ANC) and skilled birth attendance (SBA) during childbirth. An important component of the strategy was community engagement and sensitization. Furthermore, recognizing that unsafe abortion was an important cause of maternal mortality, the GoE expanded the circumstances under which safe abortion care could be provided. The law on safe abortion services authorizes the termination of pregnancy in certain circumstances including in cases of rape, incest, and fetal impairment, to save the life of the woman, if the if the woman has a physical or mental disability, or if she is under 18 years of age [14]. It could be provided without requiring proof of age or parental consent [13, 14].

The GoE has demonstrated a high level of political commitment in combating HIV/AIDS, as evidenced by establishment of the National AIDS control programme since 1987 under the Ministry of Health. Stemming from the lack of guidance on intervention activities undertaken by the government, NGOs and [15]. A Framework for the National Response to HIV/AIDS was adopted, outlining priority interventions for promoting and distributing condoms among other strategic issues with a multisectoral approach, engaging the public and private sectors, including NGOs, community-based organizations (CBOs) and faith-based organizations [16]. The GoE formulated a national policy on viral-load monitoring and a system to carry out quality assessment at the health facility-level on Antiretroviral Therapy (ART) and AIDS in 2003 and by decentralizing the free Highly Active Antiretroviral Treatment (HAART) in public facilities and private clinics and hospitals in 2005 [17]. To address the specific needs of adolescents and young people, the GoE organized a “National Youth Consultation on HIV/AIDS, Sexual and Reproductive Health in Ethiopia” in 2003, which informed the development of the National Youth Policy in 2004 and the Youth Development Package in 2006 [18]. By 2019, the GoE formulated and executed a national policy on HIV self-testing (HIV-ST), as a complementary approach to the existing facility-based HIV Testing Services, with the aim of reaching previously untested, hard-to-reach and test-averse populations [19]. Further to this, the GoE has developed four National Multisectoral Strategies. The strategic issues focus on capacity building, community mobilization and empowerment, integration with health programs, leadership and mainstreaming, coordination and networking and focus on special target groups with a multi-sectoral approach. The National HIV/AIDS Strategic Plan for 2021–2025 calls for self-testing (HIVST) to be introduced through social marketing and private providers to the general population on fee-payment basis with instructions for people testing positive to obtain confirmatory tests at health facilities located closest to them [20].

The GoE developed a National Strategy and Action Plan on Harmful Traditional Practices against Women and Children in 2013 [21]. The plan contained specific targets for the reduction of child marriage as part of broader targets for gender equality It also reiterated commitments to eliminate child marriage by 2025 in the Ethiopian Girl Summit that took place in 2014 [22]. The GoE’s leadership in this area stimulated and supported NGO-led efforts in the country. For example, Berhane Hewan (“Light for Eve”) a project which pilot tested a package of interventions between 2004–2006 to address child marriage was led by the Ethiopia Ministry of Youth and Sports, the Amhara Regional Bureau of Youth and Sport and in collaboration with the Population Council. It involved various stakeholders, and included multiple components to delay child marriage, including the engagement of out-of-school girls in non-formal education, livelihoods training, and the provision of support for girls to remain in school [23]. Building on the evidence base from the pilot project, the intervention was continued and expanded between 2010–2016 [23].

The National Strategy and Action Plan on Harmful Traditional Practices against Women and Children in 2013 also contained specific targets for the reduction of FGM/C as part of broader targets for gender equality [21]. Further, the GoE has established specific legal measures and actions to address violence against women and girls, including the revision of the Family Law in 2000 and the Penal Code in 2005 [24]. The GoE has also put in place institutional mechanisms at the federal and regional levels, including the establishment of the Ministry of Women, Children and Youth Affairs (MoWCYA), Child and Women Protection Units within the various police units, and a Special Bench for violence against women within the federal criminal court [25].

To conclude, over the last two decades, the evolving political, economic and social context of the country have placed some aspects of ASRHR high on the health and development agendas. The formulation and application of laws, policies, strategies and programmes have contributed to effective action in a number of areas, but there are still notable gaps and areas of weakness in translating them into action. More needs to be done to communicate these laws and policies, and to resource and manage the implementation of the strategies and programmes, and to carry the wider society along in this shared effort.

## Substantive changes have occurred in a number of health outcomes, harmful practices, health behaviours, health service use, and social determinants in the first two decades of the twenty-first century

### A substantial though uneven decline occurred in the levels of child marriage

According to a 2018 UNICEF report on child marriage trends, the overall prevalence rate of child marriage in Ethiopia has dropped by a third in the last 10 years [26]. However, geographical variations in child marriage exist with different levels of prevalence and rates of decline across the regions. With regard to prevalence, for example, in 2016, Afar had the highest rate of child marriage (67.4%), followed by Benishangul (50.3%) [5]. With regard to rates of decline, for example, in 2005, the rate of child marriage in Amhara (74.4%) was three times higher than that in Addis Ababa (13.7%) [4]. By 2016, the rate had decreased by 31.4 percentage points in Amhara and by 5.9 percentage points in Addis Ababa. Despite the progress, the prevalence of child marriage is still quite high at 40.3% in 2016 [5].

The median age at first marriage varies by region from 15.7 years among women in Amhara to 23.9 years in Addis Ababa in 2016, with an increase of 1.5 years in Amhara and 2 years in Addis Ababa since 2005 [4, 5] and in 2013, women aged 20–24 years were marrying about 3 years later than their counterparts three decades ago [27]. The proportion of married adolescent girls aged 15–19 years decreased by six percentage points from 23.2% in 2000 to 17.4% in 2016 [3, 5].

### There has been a slight reduction in adolescent childbearing

The overall proportion of adolescents that have begun childbearing has decreased by 3.8 percentage points between 2000 and 2016, which could be in part due to the decrease in the proportion of adolescents that ever-had sex and early sexual debut. However, there are large regional variations; the rate ranges from 3% in Addis Ababa in 2016 to 23.4% in Afar in the same year [3, 5]. The variation between married and unmarried adolescents remains quite large over time with a decrease of 1.8 percentage points in the proportion of married adolescent girls with repeat pregnancies (see Table 1). In Ethiopia, as in other countries with similar social, economic and cultural contexts, girls are typically expected to bear a child soon after marriage [28]. The persistently high proportion of married adolescents who have at least one child clearly reiterates the role of societal norms in childbearing soon after marriage [28, 29]. The low rates of child marriage in some regions in Ethiopia, coupled with low rates of childbearing in the same regions point to an intersectionality between child marriage and childbearing. For example, Addis Ababa has one of the lowest rates of child marriage and of childbearing.

**Table 1:** Adolescent childbearing and sexual debut (2000–2016)

	2000	2005	2011	2016	Absolute percentage change (2000–2016)	Relative percentage change (2000–2016)
% of Adolescents that have begun childbearing						
Married	63.2	68.2	58.4	59.5	− 3.7	− 5.9
Unmarried	2.0	2.3	1.5	2.6	0.6	+ 30.0
Children ever born (married adolescents aged 15–19						
At least one child	39.9	38.4	36.3	41.9	2.0	5.0
At least two children	7.8	13.9	9.5	6.0	− 1.8	− 23.1
Ever had sex						
Females	30.7	27.4	24.2	24.6	− 6.1	− 19.9
Males	15.4	7.2	7.8	8.1	− 7.3	− 47.4
Sexual debut by age 15						
Females	13.5	11.1	7.1	6.3	− 7.2	− 53.3
Males	5.1	1.7	1.2	0.8	− 4.3	− 84.3

**Fig. 1:**
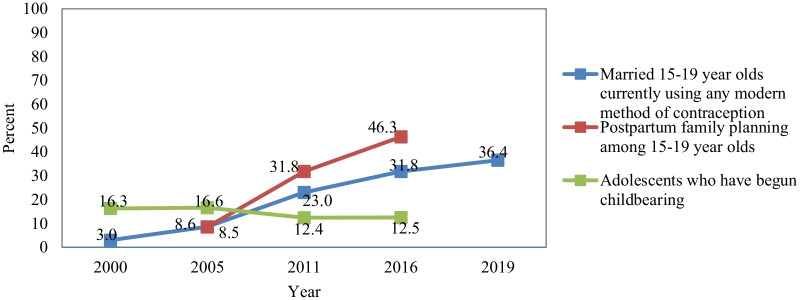
Adolescent childbearing and modern contraceptive prevalence among adolescent girls in Ethiopia (2000–2019)

The decrease in adolescent childbearing could also be partly attributed to an increase in the use of modern contraceptives. The modern contraceptive prevalence rate (mCPR) among adolescents in Ethiopia currently stands at 36.4% [30]. Figure 1 shows a sharp increase in the proportion of married adolescent girls using contraception and a percentage point increase of 38% in the use of post-partum contraception between 2005 and 2016.

Finally, there has been a reduction in the equity gap in modern contraceptive use among married adolescent girls with a positive average annual rate of change of 10% and 12% across all education levels and urban–rural areas respectively [31]. The evidence points to a clear association between increased levels of education and reductions in early marriage and early childbearing [32]. The median age at first marriage increases with increasing education, from 16.3 years among girls with no education to 24 years among girls with more than a secondary education [5]. The median age at first birth has seen an increase of 0.5 years among those with no education and 1.6 years among those with more than secondary education, between 2000 and 2016. This is in contrast to a slight decrease of 0.1 year among those with more than secondary education [3, 5].

**Fig. 2:**
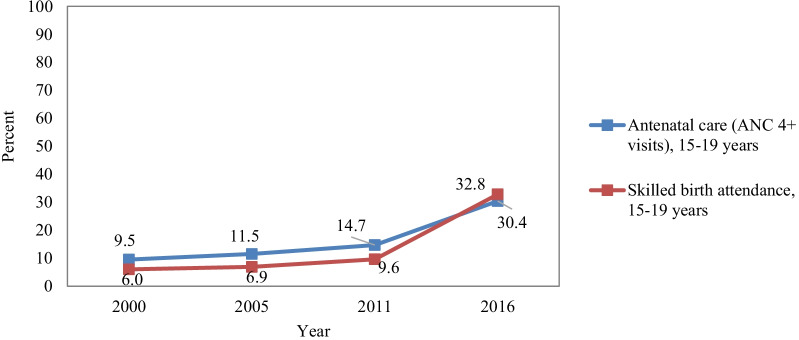
Health service uptake among adolescents in Ethiopia (2000–2016)

Although SRH service uptake rates are lower in adolescents compared to the general population, they have increased over the last 20 years. ANC visits of four or more (ANC4+) and the use of SBA among adolescents have increased over time, yet the levels of use are still low (see Fig. 2) [3–6]. Further, there are enormous geographical variations, with the use of SBA among adolescent girls aged 15–19 years ranging from 25% in the Somali region to 88% in Addis Ababa in 2016 [5]. Finally, women who married later are at least twice as likely to have delivered their most recent baby in a health facility compared to women married before age 15 [29].

The overall national Maternal Mortality Ratio (MMR) has decreased from 673 in 2005 to 412 deaths per 100,000 live births in 2016 [4, 5]. Household survey data suggest that the proportion of female deaths that are pregnancy-related among adolescent girls aged 15–19 has decreased by 20% relative points between 2011 and 2016, although the progress was non-monotonous between 2000–2016 with the proportion decreasing from 19% in 2000 to 12% in 2005, increasing to 22% in 2011 [3–6].

Although there is a paucity of adolescent-specific abortion data in the country, the available data point to an increase in adolescents seeking abortion services in health facilities. In 2008, the abortion rate for adolescents aged 15–19 was 11 per 1000 women of that age, with 9% of pregnancies ending in abortion [33]. This figure rose to 19.6 per 1000 women aged 15–19 in 2014, with 20% of pregnancies ending in abortion [33]. The estimated incidence of legal and clandestine abortions based on 2014 data shows that adolescents who were sexually active (< 12 months) had the highest proportion of legal abortions (64%) when compared to other age groups [31]. For example, a study in one hospital in Ethiopia found that women under the age of 19 years were twice as likely to have a second trimester abortion compared to their older counterparts [34]. The higher abortion rate among adolescents suggests barriers in access to and use of contraceptive services, with at least one-third of pregnancies occurring among 13–16-year-olds being unintended [35]. There is no evidence that adolescents are more likely than older women to have clandestine abortions. However, critical gaps remain; although abortion is legal under specific circumstances, an estimated one-third of adolescent abortions are clandestine and potentially unsafe, these are mainly due to lack of information about location and availability of services, pressure from families and communities, poorly equipped facilities, absent providers, and a weak referral system [32].

### There is some indication that the HIV infection rate in adolescents is declining. However, the numbers of adolescents using condoms and seeking HIV testing show a mixed picture

**Fig. 3:**
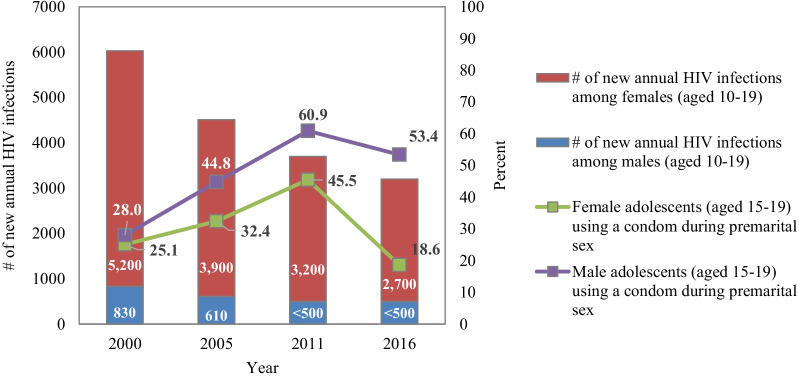
HIV and condom use among adolescents in Ethiopia (2000–2016)

The estimated number of new annual HIV infections among adolescents aged 10–19 decreased between 2000 and 2018, from 6000 to 3000 cases among both boys and girls [36]. However, there are clear disparities in terms of gender. Similarly, use of condoms among adolescent during premarital sex shows a mixed picture, with a steady increase between 2000 and 2011, but a sharp decline in 2016 [4, 6] (see Fig. 3).

HIV testing among sexually active (< 12 months) adolescent girls who received their results showed good progress between 2005 and 2016 with a percentage point increase of 24%; however, progress slowed between 2011 and 2016, with only a modest percentage point increase of 6%. Meanwhile, HIV testing among sexually active (< 12 months) adolescent boys showed a decline of 33 percentage points between 2011 and 2016 [4, 6].

The actions by the GoE to rapidly scale-up HIV treatment and multi-sectoral approach to HIV/AIDS response are attributed to the prevention of large numbers of new infection in the country [18]. However, the mixed picture on HIV testing and condom use can be linked to low condom distribution to key populations, as a result of repeated stock-outs that could not be alleviated even with some redistribution schemes and diversion of free condoms for commercial purposes [18]. Delays in finalizing the condom strategy and implementation guidelines likely contributed to this [18].

### FGM/C rates have shown a promising decline

The proportion of adolescent girls that have undergone FGM/C has sharply decreased by 24 percentage points (from 71 to 47%) between 2000 and 2016 [3, 5]. However, at least 28% of adolescent girls aged 10–14 years had experienced FGM/C in 2016, with Afar having the highest rate at 86% and Tigray the lowest rate at 14%. The high rates of FGM/C in some regions are upheld by traditions that see FGM/C as a means of social acceptance by symbolizing preservation of virginity and improving marriage prospects [37, 38]. However, the overall progress in decreasing FGM/C in the country is attributed to the community involvement, coupled with a legal code that criminalizes perpetuators of such practices [25].

## Levels of reported gender-based violence against girls and women remain high, but attitudes towards wife beating have changed

At least 33% of ever married adolescent girls have ever experienced physical, sexual or emotional violence committed by their husband or partner [5]. As elsewhere in the world, it is likely that many cases of GBV go unreported. The last two decades have seen a significant change in attitudes towards wife beating, with a percentage point decrease of 22% and 49% among girls and boys respectively condoning such violence between 2000 and 2016 [3–6].

Overall, ASRHR in Ethiopia has achieved substantial progress over the last two decades; however, there has been more progress in some areas than in others. Even where there has been progress, it has been inequitable with enormous variations by geographic location, and by education and wealth levels [39].

## As we look to the next 10 years, Ethiopia must build on the progress made, and move ahead understanding and overcoming challenges and making full use of opportunities

### What are the opportunities and challenges for moving ahead?

In the 25 years since the ICPD, Ethiopia has used available opportunities to accelerate progress on ASRHR and created some new ones. Specifically, the country has demonstrated committed leadership on ASRHR. It has established an enabling legal and policy environment and put in place sound health and gender strategies. These strategies have been well resourced, managed and implemented. Different government sectors have been involved in this work. Further, the Ministry of Health has built partnerships at national and sub-national levels with academic institutions, NGOs and professional associations of public health professionals and clinicians. And at the local level, it engaged extension workers from the communities in which they would serve. These workers were aware of the prevailing context and were trained and supported to engage in dialogue to challenge norms such as those related to child marriage and the low utilization of contraception or maternal health services [12]. Finally, it encouraged the active engagement of youth-serving NGOs and youth-led networks to promote their leadership and engagement [40].

At the same time, a number of challenges for accelerating progress on ASRHR persist. As noted in the previous section, there is some skepticism of the value of investing in adolescent health and development, and limited and uneven awareness of the enabling laws, policies, strategies and plans among both the frontline workers who are mandated to carry them out, and among the communities they are meant to benefit. Likewise, the implementation of these laws, policies, strategies and plans has been hampered by lack of both human and system capacity. As a result, implementation in some places, especially in rural areas has been fragmented and poorly coordinated, and in addition there is limited health system readiness to adequately respond to the needs and preferences of adolescents. Following from this, the progress that has been made in some aspects of ASRHR has been uneven across the country, with evident equity gaps. Finally, although some social norms appear to be changing, many other norms and attitudes, such as those that stigmatize premarital sexual activity, encourage early marriage, and value large family size persist [34, 37, 38]. This is coupled with resistance in some quarters to certain aspects of ASRHR such as comprehensive sexuality education.

In the past two decades, there have been several AYSRH programs implemented all over the country. However, these programs have been implemented with limited, and often token engagement of young people. These programmes could have been more effective if the key stakeholders, adolescents and young people were allowed to take part in design, planning, implementation and monitoring and evaluation. Further, the indirect effects of COVID-19 and the conflicts and internal displacements in the country could contribute to a stalling or even a reversal of the gains described in this commentary [41]. For example, inter-communal tensions along the Southern Nations, Nationalities, and People’s (SNNP)-Oromia regional boundaries, violence in Benishangul-Gumuz region and civil strife in the Tigray region have likely negatively affected health and development programmes including those of ASRH [42]. Similarly, natural disasters in Afar, Amhara, Gambella, Oromia, SNNP and Somali regions have likely increased the vulnerability of populations in these regions to health and social problems, and in adolescents this can lead to problems such as unintended pregnancies and GBV [43].

## With these opportunities and challenges in mind, what key actions must be taken to accelerate progress on ASRHR?

Ethiopia must make full use of the existing political support for ASRHR policies and programmes and sustain this support in the next stage of strategy development, while working to increase social support. In the last two decades, the Federal Ministry of Health of Ethiopia demonstrated commitment and leadership to addressing adolescents’ needs. This is particularly exemplified by the 2016–2020 Adolescent and Youth Health (AYH) Strategy, the Reproductive Health Strategy and the National Strategy and Action Plan on Harmful Traditional Practices. It is also exemplified by the establishment of an AYH case team with four designated personnel under the MNCH Directorate and the assignment of AYH focal persons at regional, zonal and woreda levels. This political support has been central to the advancement of ASRHR. Moving forward, this must be sustained, especially to ensure that ASRHR is adequately addressed in the next iteration of the AYH strategy.

In contrast, there is still lack of widespread social support for some aspects of ASRHR such as the provision of safe abortion care. Premarital sexual activity is not widely acknowledged; young couples face pressure to have a child shortly after marriage; child marriages and FGM/C while declining, continue to occur; and parent–child communication on SRH is limited. Therefore, moving forward, there is a need to step up efforts to challenge and change social norms affecting ASRHR by engaging religious and community leaders, parents and families, and adolescents themselves more actively, including through community dialogue on traditional values that negatively affect the health of adolescents, particularly girls. Increased school enrolment provides a window of opportunity to advocate for comprehensive sexuality education that challenges societal norms in making SRHR information accessible to adolescents.

Ethiopia must strengthen investment in and financing of interventions to meet the SRH needs of adolescents. While the country has taken great strides in the last two decades to ensure the provision of free maternal and newborn health services in public health facilities, the provision and uptake of some other interventions are hampered by the lack of financing and are not covered by the national health insurance scheme. Moving forward, there is a need to advocate for increased allocation of external and domestic funding for these interventions, taking into account the short- and long-term health and social outcomes as well as the costs of inaction for current adolescents and future generations. Importantly, this must include financial investments in the health sector, as well as other sectors which have a role to play in promoting ASRHR.

The opportunity presented by the country’s tremendous progress must be seized to create an enabling legal and policy environment by ensuring that laws and policies are appropriately communicated, applied and monitored. As noted above, there is a low level of awareness about these laws, policies, and strategies among health care providers, teachers, parents, adolescents, and the community at large. Likewise, implementation of these laws and policies is hindered by the limited capacity of the relevant authorities, including law enforcement bodies. Moving forward, there is a need to step up efforts to inform all relevant stakeholders of the laws and policies in place, advocate for their proper implementation, and monitor them to ensure that they are applied appropriately and effectively.

Further, Ethiopia must continue to ensure its strategies are evidence-based and extend the availability of age-disaggregated data on SRHR. The 2016–2020 National AYH Strategy was successful in incorporating evidence-based interventions and delivery approaches, including for ASRHR. Moving forward, it will be important to ensure that the next iteration of the strategy is also grounded in the most up-to-date evidence available. Similarly, initial steps have been taken in gathering age and sex disaggregated data in health facilities in some areas of ASRH. This needs to be extended to all indicators relevant to adolescent health within and outside SRH (i.e., to track progress on the health outcomes addressed in AYH strategy), as well to ensure age and sex disaggregated reporting of this data.

Ethiopia must double down with renewed focus to ensure that its strong national strategies are managed effectively, and interventions delivered at scale, with quality and equity. In 2016, the National AYH Strategy rapidly increased in scope without sufficient expansion of its implementation mechanisms, resources, and infrastructure. As a result, there has been a lack of adequate coordination, inadequate resource allocation and mobilization, and implementation capacity, resulting in a diluted focus on ASRHR [44]. Moving forward, this calls for critically re-evaluating the readiness of the health, education and other systems at all levels (national, regional, zonal/district to facility) to translate strategies into programmatic action in line with a standards-driven approach to improve the quality of SRH services for adolescents. Likewise, it may require starting with a more limited scope and progressively expanding the focus as implementation improves. Specifically, this will require attention to five areas: promoting improved multi-sectoral coordination; ensuring adequate health care provider (and other relevant frontline worker) competencies and attitudes by moving beyond one-off, off-site trainings; tailoring interventions and delivery approaches through sub-national plans and strategies to account for diverse health situations and socio-economic contexts; committing to learn-by-doing on an ongoing basis; and making every effort to address the needs and vulnerabilities of those who are marginalized and could be left behind.

While there is some engagement of adolescents and young people in programme design, planning, implementation monitoring, and evaluation, this is not always fully meaningful, as already noted [40]. Moving forward, it is important that adolescent participation is institutionalized as an essential element of all programmes intended to benefit them, in particular implementing the guidelines on youth engagement launched by the Ministry of Health. This should be complemented with technical and financial support for adolescent and youth-led organizations.

In light of the current COVID-19 pandemic, it will be particularly important to keep an ear to the ground to understand how COVID-19 (and the responses put in place to address it) is impacting adolescents and address their needs with responses that are sufficiently flexible to remain relevant and appropriate as the pandemic evolves [40]. Further, it is important for peace and harmony to be restored and that the special needs of adolescents and young people are taken into account in responses in humanitarian settings.

## Data Availability

The datasets generated and/or analysed during the current study are available from the Ethiopia Demographic and Health Survey: https://dhsprogram.com/data/.

## Abbreviations

AYH: Adolescent and Youth Health
AIDS: Acquired Immunodeficiency Syndrome
ANC: Antenatal care
ART: AntiRetroViral Therapy
ASRHR: Adolescent sexual and reproductive health and rights
FGM/C: Female genital mutilation/cutting
GBV: Gender-based violence
GDP: Gross Domestic Product
GoE: Government of Ethiopia
HEW: Health Extension Workers
HAART: Highly Active Antiretroviral Treatment
HIV: Human Immunodeficiency Virus
HIVST: HIV self-testing
HMIS: Health Management Information Systems
ICPD: International Conference on population and development
MCPR: Modern contraceptive prevalence rate
MMR: Maternal mortality rate
MNCH: Maternal and Child Health
MoWCYA: Ministry of Women, Children and Youth Affairs Offices
NGO: Non-Governmental Organisation
SBA: Skilled birth attendance
SDG: Sustainable Development Goal
VAW: Violence against women
