# Correction to: ASRHR in Ethiopia: reviewing progress over the last 20 years and looking ahead to the next 10 years

**DOI:** 10.1186/s12978-022-01474-y

**Published:** 2022-08-04

**Authors:** Elsie Akwara, Kereta Worknesh, Lemessa Oljira, Lulit Mengesha, Mengistu Asnake, Emiamrew Sisay, Dagem Demerew, Marina Plesons, Wegen Shirka, Azmach Hadush, Venkatraman Chandra-Mouli

**Affiliations:** 1 Department of Sexual and Reproductive Health and Research/Human Reproduction Programme, World Health Organization, Geneva, Switzerland; 2 Adolescent and Youth Health and Development Programme, Pathfinder International, Addis Ababa, Ethiopia; 3 School of Public Health, College of Health, College of Health and Medical Sciences, Haramaya University, Addis Ababa, Ethiopia; 4 Youth Council, Talent Youth Association, Addis Ababa, Ethiopia; 5 Pathfinder International, Addis Ababa, Ethiopia; 6 Federal Ministry of Health, Addis Ababa, Ethiopia; 7 Ethiopia Get Up Speak Out Alliance, Addis Ababa, Ethiopia; 8 Child and Adolescent Health, World Health Organization, Addis Ababa, Ethiopia; 9 Health Systems Unit, World Health Organization, Lusaka, Zambia

## Correction to: Reproductive Health (2022) 19:123 https://doi.org/10.1186/s12978-022-01434-6

After publication of this article [1], the authors reported that the author name ‘Venkatraman Chandra Mouli’ was incorrectly written as ‘Venkatraman Chandra Moulli’. Also, the author name ‘Lamessa Oljira’ was incorrectly written as ‘Lamessa Oljiira’.

Moreover, in the heading ‘Substantive changes have occurred in a number of health outcomes, harmful practices, health outcomes, health behaviours, health service use, and social determinants in the first two decades of the twenty-first century’, remove “health outcomes”, since it appears twice.

The original article [1] has been corrected.
